# Microscopic photoelectron analysis of single crystalline LiCoO_2_ particles during the charge-discharge in an all solid-state lithium ion battery

**DOI:** 10.1038/s41598-019-48842-6

**Published:** 2019-08-28

**Authors:** Keishi Akada, Takaaki Sudayama, Daisuke Asakura, Hirokazu Kitaura, Naoka Nagamura, Koji Horiba, Masaharu Oshima, Eiji Hosono, Yoshihisa Harada

**Affiliations:** 1 Institute for Solid State Physics, The University of Tokyo, Kashiwa, Chiba 277-8581 Japan; 2 Research Institute for Energy Conservation, National Institute of Advanced Industrial Science and Technology, Tsukuba, Ibaraki 305-8568 Japan; 3 AIST-UTokyo Advanced Operando-Measurement Technology Open Innovation Laboratory (OPERANDO-OIL), National Institute of Advanced Industrial Science and Technology (AIST), Kashiwa, Chiba 277-8565 Japan; 4 Research Center for Advanced Measurement and Characterization, National Institute for Materials Science, 1-2-1, Sengen, Tsukuba, Ibaraki 305-0047 Japan; 5 PRESTO, Japan Science and Technology Agency, 4-1-8, Honcho, Kawaguchi, Saitama 332-0012 Japan; 6 Institute of Materials Structure Science, High Energy Accelerator Research Organization (KEK), 1-1 Oho, Tsukuba, 305-0801 Japan; 7 Synchrotron Radiation Research Organization, The University of Tokyo, Bunkyo-ku, Tokyo 113-8586 Japan

**Keywords:** Batteries, Characterization and analytical techniques

## Abstract

We report synchrotron-based *operando* soft X-ray microscopic photoelectron spectroscopy under charge-discharge control of single crystalline LiCoO_2_ (LCO) particles as an active electrode material for an all solid-state lithium-ion battery (LIB). Photoelectron mapping and the photoelectron spectrum of a selected microscopic region are obtained by a customized *operando* cell for LIBs. During the charge process, a more effective Li extraction from a side facet of the single crystalline LCO particle than from the central part is observed, which ensures the reliability of the system as an *operando* microscopic photoelectron analyzer that can track changes in the electronic structure of a selected part of the active particle. Based on these assessments, the no drastic change in the Co 2*p* XPS spectra during charge-discharge of LCO supports that the charge-polarization may occur at the oxygen side by strong hybridization between Co 3*d* and O 2*p* orbitals. The success of tracking the electronic-structure change at each facet of a single crystalline electrode material during charge-discharge is a major step toward the fabrication of innovative active electrode materials for LIBs.

## Introduction

Development of high-performance lithium-ion batteries (LIBs) with large energy density, high power performance, high charge-discharge cycle stability, and high safety is strongly demanded to promote the use of clean energy devices, such as electric vehicles, to build a sustainable low-carbon society^1^. In the development of negative electrode materials for LIBs, strategies for the materials design have been established and metal lithium or silicon-lithium alloys have attracted much attention as high capacity materials^2,3^. However, strategies for such innovative materials to be used at the positive electrode have not been established because there are a lot of unknown charge-discharge mechanisms that occur at the positive electrode^4–8^. To overcome this situation, researchers have undertaken cutting-edge analytical techniques to further understand these positive electrode materials and to create a strategy for materials design. Various kinds of analytical techniques, like two-dimensional nuclear magnetic resonance spectroscopy^9–11^, annular bright-field and high-angle annular dark field scanning transmission electron microscopy^12,13^, and micro-Raman spectroscopy^14–16^, have been widely used in the research of battery materials, and these methods have unveiled a lot of information on the microscopic structures in LIBs, such as the partial structure of organic compounds at the solid-electrolyte interface, distributions of each element like lithium, metal, and oxygen, and cross-sectional mapping information in a large area of the coated electrode films.

For the positive electrode materials of LIBs, lithium insertion and extraction change the crystal structure of the host crystals. The crystal-structural change is triggered by the changes in the electronic structure of the host material during the Li insertion/extraction process^17^. In addition, lithium ions diffuse into the host crystals along a direction that is dependent on the crystal structure. For example, diffusion of lithium ions in LiFePO_4_ (olivine-type), LiCoO_2_ (LCO, layered rock salt), and LiMn_2_O_4_ (spinel-type) is one-dimensional, two-dimensional, and three-dimensional, respectively^18–20^. To understand the above-mentioned complex phenomena, analytical techniques using synchrotron radiation X-rays have enabled a major advance: hard X-rays having a short wavelength can be used to clarify the crystal structure^21,22^ and soft X-ray spectroscopies, which cover the 3*d* transition-metal *L* and oxygen *K* edges, are advantageous to obtain element-specific electronic-structure information on the host material^23–28^. Concerning the measurement area on the electrode materials, the recent development of X-ray optics has enabled a focusing of the X-ray beam down to the several tens of nanometer scale^29–31^. This has enabled us to observe various micro areas of the microcrystals of electrode materials and understand the stability of each micro region^6,32,33^. More important has been the rapid growth of the state-of-the-art *operando* X-ray measurements, where X-ray progress can be performed during voltage/current operation of the battery^6,23,27,34–36^. All of the characteristics of synchrotron X-ray spectroscopies are essential for accurate observation and detailed understanding of the diffusion and domain wall kinetics of the intercalation electrode materials and even more complex charge-discharge mechanisms, including redox potential, capacity, and cycle stability of electrode materials.

Here, we report the *operando* analysis of X-ray photoelectron microscopy by using an all-solid-state lithium-ion battery cell originally developed for the 3DnanoESCA station at the University of Tokyo Synchrotron Radiation Outstation beamline BL07LSU in SPring-8^29^. A single crystalline hexagonal plate of LCO, with a size of around 10 µm, was fabricated by a flux method as the target material. LCO is suitable as a model material for the novel analysis method because LCO is a typical material for use at the positive electrode of LIBs. We achieved lithium insertion and extraction of LCO and obtained the mapping image of photoelectrons for selected energy regions at each area under potential control.

## Results and Discussion

**Figure 1:**
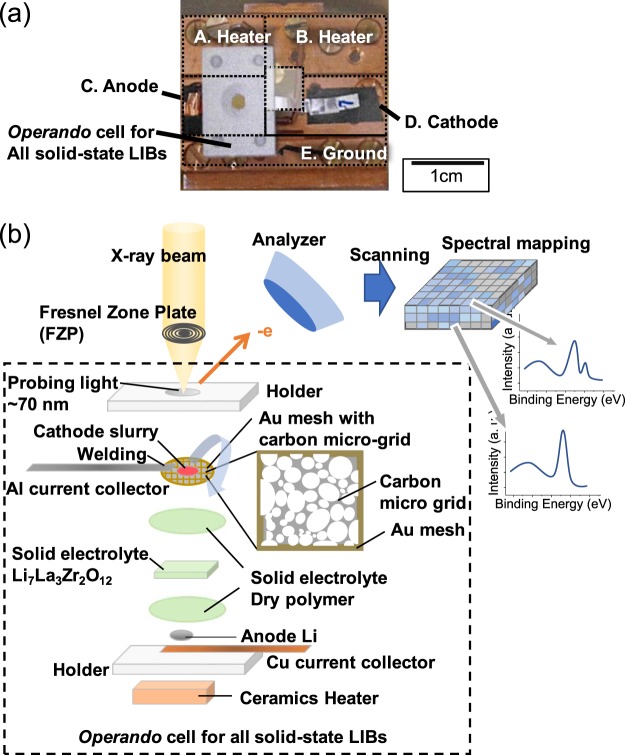
*Operando* photoelectron microspectroscopy system of 3DnanoESCA for all solid-state LIBs. (**a**) Picture of cell holder for all solid-state LIBs with five terminals for anode, cathode, ground and heaters. (**b**) Schematic details of all solid-state LIBs.

Figure 1 displays the *operando* photoelectron microspectroscopy system for the all solid-state LIBs. A photoelectron map could be obtained by scanning a soft X-ray beam focused on a sample using a Fresnel zone plate (FZP) and detecting the photoelectrons at each beam spot. An *operando* cell for the all solid-state LIBs was prepared for photoelectron microspectroscopy in a high vacuum chamber.

As the working electrode, an Au mesh with a carbon micro-grid was used for electrochemical measurements and photoelectron mapping. An Al sheet was welded to the edge of the Au mesh to establish an electrical connection to one of the five terminals of the cell holder as the cathode current collector. An LCO slurry was dropped onto the micro-grid and the dried slurry was sandwiched with a solid electrolyte. We used dilute slurry, i.e. small amount of LCO particles, for the clear observation of each isolated single crystalline particle on the microgrid. Before installation to the high vacuum chamber, a high magnification optical image was obtained to identify the X-Y coordinates of the selected single crystalline LCO particles. A Cu current anode collector, metal Li as anode material, PEO/LiTFSI solid (dry polymer) electrolyte, Li_7_La_3_Zr_2_O_12_ (LLZ) ceramics electrolyte, PEO/LiTFSI dry polymer electrolyte, and the Au mesh with LCO were all fixed by ceramics jigs. The adhesive property of PEO/LiTFSI dry polymer created a good interface between the dry polymer and the metal Li, LLZ, and LCO to decrease the interfacial resistance. The carbon micro-grid played the role of a conduction path from the Au mesh to the single crystalline LCO particles.

During the limited time of the synchrotron radiation X-ray analysis, the *operando* cell was heated by a ceramics heater to approximately 55 °C to improve the ionic conductivity of the dry polymer and LLZ, which realized a high CV sweeping rate of the cell. Terminals A and B were used to supply the current for the heating. The *operando* cell was set on the cell holder in a glove box and transferred to 3DnanoESCA without air exposure.

**Figure 2:**
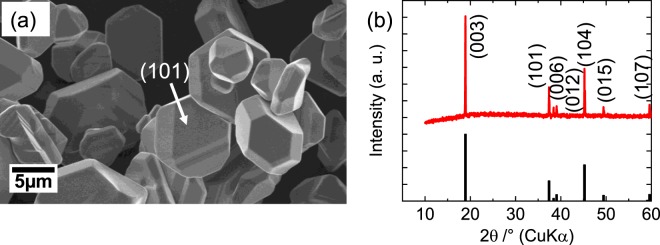
(**a**) SEM image and (**b**) XRD pattern of single crystalline LiCoO_2_ particles. XRD peaks are assigned from a ICDD data of LiCoO_2_ (ICDD-PDF No. 01-070-2685).

To discuss the relationship between Li diffusion and redox reaction of Co in the host crystal, we collected the SEM image and XRD pattern of the single crystalline LCO particles. Considering the spatial resolution of the 3DnanoESCA station, the particle size of the single crystals was tuned to approximately 10 µm to distinguish the photoemission signal from each facet. Figure 2(a) shows the SEM image of the single crystalline LCO particles. The plate morphology of a triangular facet in the (101) plane^37^ with a size of approximately 10 µm was observed. Therefore, adequate single crystalline LiCoO_2_ for *operando* measurement by the 3DnanoESCA station was fabricated by the flux method. Figure 2(b) indicates the XRD pattern of the fabricated sample and a pattern from the ICDD-PDF data of LCO (No. 01-070-2685). The XRD pattern was in excellent agreement with the ICDD pattern. The obtained lattice constants of *a* = 2.816 Å and *c* = 14.053 Å were almost identical to those of *a* = 2.816 Å and *c* = 14.054 Å in the ICDD-PDF data of LCO.

**Figure 3:**
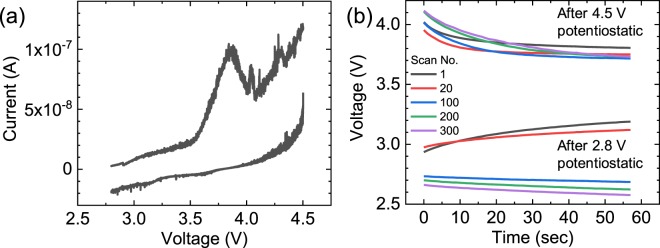
Li extraction and insertion by *operando* measurement system in 3DnanoESCA.

Cyclic voltammetry was conducted for Li extraction and insertion of the single crystalline LCO particles. Figure 3(a) shows the Li extraction up to 4.5 V (vs. Li/Li^+^) and Li insertion down to 2.8 V (vs. Li/Li^+^). A peak around 3.5–4 V was caused by an oxidation reaction of LCO with the extraction reaction of Li. During the photoemission measurements, the potential was intentionally controlled by repeating a potentiostatic mode at 4.5 V (or 2.8 V) for 3 sec and an open circuit voltage (OCV) mode for 57 sec. This repeated cycling of the modes avoided self-discharge and relaxation reactions, which might result in a significant change of the potential under equilibrium conditions after extraction/insertion of Li.

Figure 3(b) shows OCV curves after the potentiostatic mode at 4.5 V and 2.8 V for 3 sec at the 1^st^, 20^th^, 100^th^, 200^th^, and 300^th^ repetition. Immediately after sweeping the voltage to 4.5 V and 2.8 V, we had to wait for the Li extraction and insertion to finish after over 30 repetitions of the potentiostatic mode for 3 sec and OCV mode for 57 sec before each *operando* photoemission measurement, because the high rate of CV sweeping gave rise to a delayed extraction and insertion of Li. The small change of the voltage in the OCV curve (e.g., from 4.5 V to approx. 4.2 V) ensured the oxidation of LCO. After the 300^th^ repetition for the 4.5 V condition, the potential was swept down to 2.8 V to monitor the Li insertion reaction and reduction of the LCO particles. A reduction peak was not observed due to the small amount of LCO particles. Nevertheless, the OCV curves after the potentiostatic mode at 2.8 V for 3 sec indicated an almost constant potential at around 3 V up to the 20^th^ repetition, which confirmed the reduction reaction of LCO by Li insertion. After the 100^th^ repetition, the potential decreased down to approximately 2.6 V, which was the initial OCV before sweeping the voltage to 4.5 V, possibly because the repetition of many times returned the cell potential to the initial condition.

**Figure 4:**
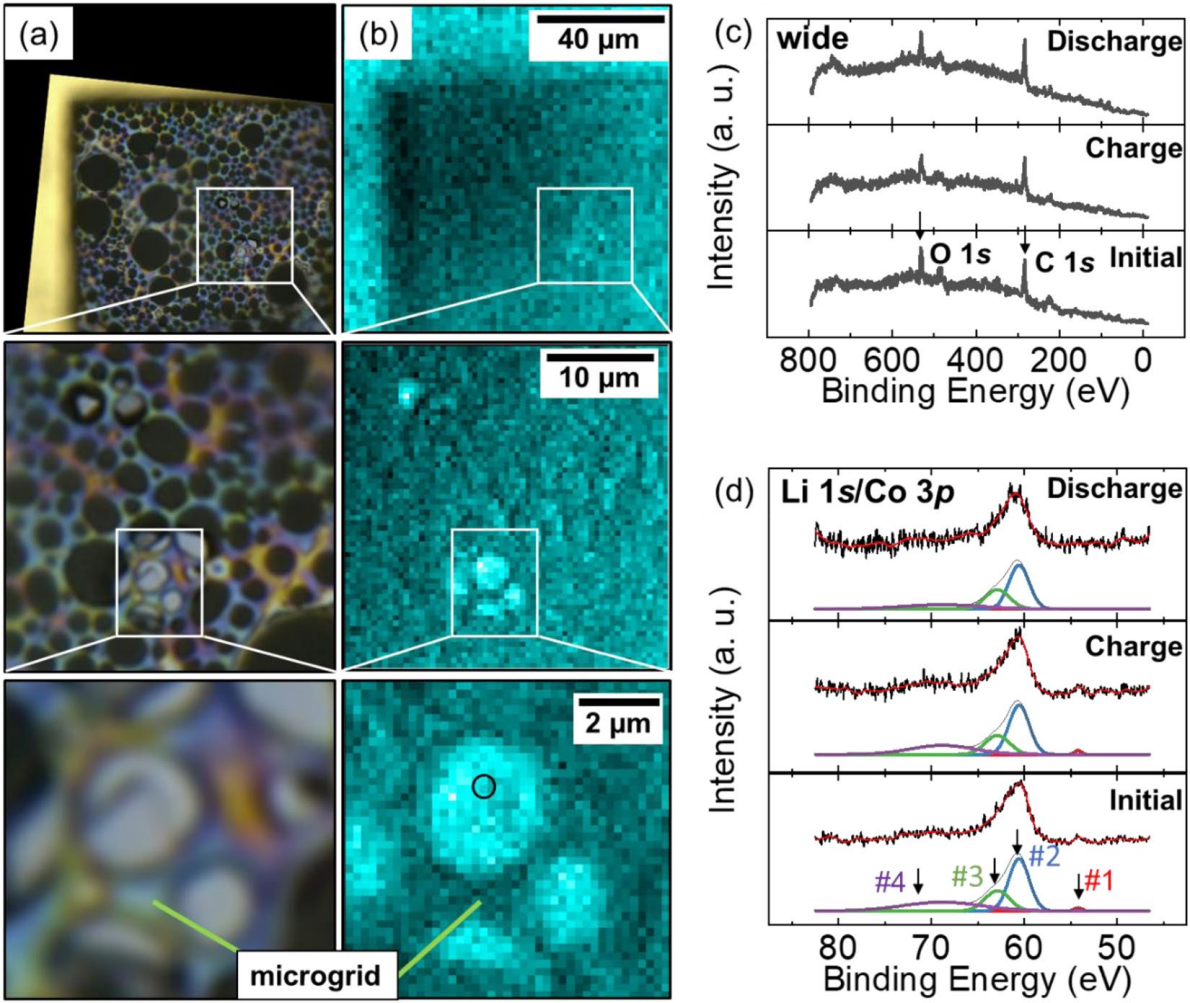
(**a**) 2D image by optical microscope and (**b**) 2D mapping of O 1*s* photoemission using the *operando* cell. Black circle indicates the measured area. (**c**) Wide and (**d**) Li 1*s*/Co 3*p* XPS spectra of LCO at initial condition, after charge and discharge condition. (#1: peak position of Li 1*s*^38^, #2: peak position of Co 3*p*_3/2_^38,39^, #3: peak position of Co 3*p*_1/2_^38,39^, #4: peak position of Co 3*p* satellite^38^).

Figure 4(a) shows the 2D image of the LCO particle obtained with an optical microscope (Keyence VHX-1000 and VH-Z500R) and Fig. 4(b) shows the corresponding 2D mapping of O 1 *s* photoemission integrated in the binding energy region of 522.5–546.5 eV. Images from a low magnification to high magnification were obtained from both microscopes. The micro-grid was overlapped on the single crystalline LCO particle in the 2D image obtained with the optical microscope while it was almost invisible in the O 1*s* photoemission image. At the center of the LCO particle, marked by a black circle in Fig. 4(b), wide scan (Fig. 4(c)) and Li 1*s*/Co 3*p* narrow scan (Fig. 4(d)) XPS spectra were obtained at the initial condition, after charge up to 4.5 V, and after discharge down to 2.8 V. While strong O 1*s* and C 1*s* sharp peaks were detected, the narrow scan XPS spectra clearly identified the Co 3*p* peak and a small contribution of the Li 1*s* peak^38^. The Co 3*p* peak profile could be fitted with three Gaussian curves for Co 3*p*_1/2_, Co 3*p*_3/2_ and satellite peaks^38,39^. Noted that the three peaks had identical energy and no drastic change of relative intensity ratios among the initial, charge and discharge conditions. It was considered that strong hybridization between Co 3*d* and O 2*p* orbitals^40^ made holes created by the charge reaction that were localized at the oxygen side, because changes in the O *K*-edge X-ray absorption spectra (XAS) for LCO upon the charge process were greater than those in the Co *L*_2,3_-edge XAS in previous *ex situ* XAS studies^40,41^. Therefore, the O 2*p* orbital should play an important role in the redox reaction rather than the Co 3*d* orbital. We could not obtain detailed information from the narrow scan O 2*p* and 1*s* spectra owing to the significant overlap of background noise by the oxygen components in the dry polymer, which were excited by a stray X-rays from outside the active area of the FZP.

In this *operando* measurement, we confirmed no drastic change of electronic structure of Co and the importance of strong hybridization of the Co 3*d* and O 2*p* orbitals. Precise discussion should be provided in the future development of the *operando* measurement system using all solid-state LIBs with oxygen free solid state electrolytes like sulphide.

**Figure 5:**
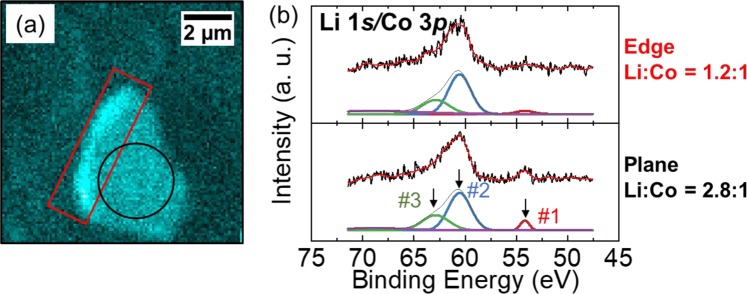
(**a**) Photoelectron-intensity-mapping of a LCO particle in the energy region of Co 3*p* and Li 1*s* and (**b**) integrate a spectra with deconvoluted peaks in two selected areas. (#1: peak position of Li 1*s*^38^, #2: peak position of Co 3*p*_3/2_^38,39^, #3: peak position of Co 3*p*_1/2_^38,39^).

Figure 5(a) shows the 2D mapping of Co 3*p* and Li 1*s* photoemission spectra integrated over the energy region from 47.5 to 71.5 eV of a single crystalline LCO particle after Li extraction by a charge reaction. The intensity of the left side of the LCO particle was higher than that of the plate area and other sides. This was because of the geometrical relationships between the photoelectron analyzer and incident beam spot on the sample. The Co 3*p* and Li 1*s* spectra integrated over the strong intensity region (red square) of the left side and weak intensity region (black circle) are shown in Fig. 5(b). Li/Co atomic ratios for the two regions can be obtained by integrating the Gaussian fits for each Co 3*p* and Li 1*s* core level and were corrected with the photoionization cross sections of Li 1*s* (0.0023 Mb) and Co 3*p* (0.061 Mb) for an incident energy of 1041 eV^42^. The Li/Co ratios were calculated to be 1.2 and 2.8 for the red square and black circle, respectively. It was considered that the lower lithium ratio of the edge region than that of the flat-center region meant that Li in the edge region was easily extracted. These results indicated that our microscopic photoelectron spectroscopy system could clearly distinguish the electronic structure of a selected part of each active single crystalline LCO particle even under *operando* condition. In addition, the spectra at each partition exhibit similar morphology when monitored by the Co 3*p* region, in spite of different ratios of Li to Co, as shown in Fig. 5(b). This result implies that the Co 3*p* spectrum is not drastically changed by the Li insertion and extraction. From these results, photoelectron mapping by the 3DnanoESCA station is useful to derive position-dependent information of the chemical and electronic states for each element.

## Conclusions

An *operando* measurement system for LIB using synchrotron radiation soft X-rays exhibits photoelectron spectra at selected micro regions and photoelectron mapping of active materials during the charge-discharge condition. Development of an all solid-state-LIB with high rate properties allows for the experiment using synchrotron radiation soft X-rays with limited time. From the *operando* analysis, important information on the charge-discharge mechanism of LCO is obtained. Generally, the redox reaction of LCO is discussed by using the formal charge of Co^3+^ ⇆ Co^4+ ^^43,44^ related by Li extraction/insertion. However, the Co spectra at the initial condition, and after charge and discharge conditions, are not changed in this *operando* measurement. It is considered that the charge-polarization might occur at the oxygen side based on the strong hybridization between Co 3*d* and O 2*p* orbitals. Thus, the importance of *operando* analysis of oxygen has been identified. Hereafter, in order to discuss the state of charge in our *operando* measurement system, a novel configuration with a working electrode made of a single crystalline particle as an active material is necessary. Moreover, we will develop this *operando* system for the analysis of electronic structure information at each facet of the single crystal and for visualization of the change in electronic structure by Li diffusion and redox reaction of elements in the host crystals.

## Method

Single crystalline LCO was fabricated by a flux method. NaCl (3.0 g), LiOH·H_2_O (0.006 mol), and Co(NO_3_)_2_·6H_2_O (0.005 mol) were placed into a Al_2_O_3_ crucible with a lid. The crucible was placed into a muffle furnace and heated at 1000 °C for 5 h in air. Finally, the samples were washed with deionized water and dried under vacuum condition.

LCO and super P Li, as an electro-conductive additive, and n-methyl-2-pyrrolidone were mixed by sonication. The slurry was dropped and dried on the Au mesh with a carbon micro-grid, which was connected to an Al current collector. The electrode was used as a working electrode of the *operando* cell, as shown in Fig. 1(b). A key point for construction of the *operando* cell is fabrication of a flat working electrode to create low interfacial resistivity for lithium ion transport and to produce an Au mesh with carbon microgrid without mechanical damage. For not only electrochemical but also mechanical reasons, to construct the *operando* cell, we needed a hard and flat solid-state electrolyte. Thus, we used the Li_7_La_3_Zr_2_O_12_ (LLZ) plate electrolyte. The interfacial resistivity between LCO and LLZ is high due to the point contact. Therefore, dry polymer electrolytes are used to decrease the interfacial resistivity.

Preparation of the *operando* cell and setting on the five-terminal folder was conducted in an Ar-filled glovebox and transferred to the 3DnanoESCA station using a transfer vessel without air exposure. Two terminals of the holder were used for the ceramics heater, as shown in Fig. 1(a). For removing water in the dry polymer of the *operando* cell, the cell was heated to approximately 55 °C in the high vacuum chamber of the 3DnanoESCA station. After that, heating was conducted during measurement to increase the ionic conductivity of both the solid-state electrolytes of LLZ and dry polymer.

For photoelectron microspectroscopy measurements on the 3DnanoESCA station at BL07LSU in SPring-8, an incident X-ray (1000 eV) was focused by a Fresnel zone plate and an order-sorting pinhole aperture of 80 μm was used. Photoelectrons were detected by a modified angle-resolved photoelectron spectrometer (VG Scienta R3000-EWAL) with a pass energy of 200 eV. In the measurement of the initial condition, the terminal of a working electrode (LCO electrode) was grounded. After the measurement of the initial condition, terminal C (counter electrode of Li metal) and terminal D (working electrode) were connected to a potentiostat-galvanostat (solartron 1287) for the electrochemical measurements. A mapping image of the scanning photoelectron microscopy was obtained by the O 1 *s* core level spectra. Selected specific micro-area photoelectron spectra were measured at the initial condition, and after charge and discharge based on this O 1*s* mapping image. Finally, the mapping images of the energy region of Li 1*s* and Co 3*p* core levels were obtained in the same LCO particle.
